# Damage Evolution of Hot Stamped Boron Steels Subjected to Various Stress States: Macro/Micro-Scale Experiments and Simulations

**DOI:** 10.3390/ma15051751

**Published:** 2022-02-25

**Authors:** Hao Zhang, Guoqiang Liu, Ning Guo, Xiangbin Meng, Yanbin Shi, Hangqi Su, Zhe Liu, Bingtao Tang

**Affiliations:** 1 School of Mechanical & Automotive Engineering, Qilu University of Technology (Shandong Academy of Sciences), Jinan 250353, China; zhanghaosdly@163.com (H.Z.); guoning19891227@163.com (N.G.); mengxiangbin84@163.com (X.M.); syb@qlu.edu.cn (Y.S.); l2434996804@163.com (H.S.); 2 Shandong Institute of Mechanical Design and Research, Jinan 250103, China; 3 Jinlei Technology Co., Ltd., Jinan 271105, China; liuzhe@jinleiwind.com

**Keywords:** bainite/martensite (B/M) decohesion, damage evolution, constitutive equation, ductile fracture

## Abstract

Hot stamping components with tailored mechanical properties have excellent safety-related performance in the field of lightweight manufacturing. In this paper, the constitutive relation and damage evolution of bainite, martensite, and mixed bainite/martensite (B/M) phase were studied. Two-dimensional representative volume element (RVE) models were constructed according to microstructure characteristics. The constitutive relations of individual phases were defined based on the dislocation strengthening theory. Results showed that the damage initiation and evolution of martensite and bainite phases can well described by the Lou-Huh damage criterion (DF2015) determined by the hybrid experimental–numerical method. The calibrated damage parameters of each phase were applied to the numerical simulation, followed by the 2D RVE simulations of B/M phase under different stress states. To study the influence of martensite volume fraction (Vm) and distribution of damage evolution, the void nucleation and growth were evaluated by RVEs and verified by scanning electron microscope (SEM). Three types of void nucleation modes under different stress states were experimentally and numerically studied. The results showed that with the increase of Vm and varying martensite distribution, the nucleation location of voids move from bainite to martensite.

## 1. Introduction

Since ultra-high strength steel (UHSS) can save fuel consumption by reducing vehicle weight and improving collision performance, it has been widely used in the automobile industry. In the past decades, due to the increasing demand for high strength body-in-white components, the hot stamping process has been spread widely [1]. During the hot stamping process, the boron steel is heated to a temperature above the austenitizing temperature, maintained and transferred to a press, where a cold die is used to obtain parts with “customized” mechanical properties. If the cooling rate of boron steel 22MnB5 blank is greater than the critical value [2], the austenite–martensite transformation would take place, leading to a tensile strength up to 1500 MPa, while the elongation would be less than 7%. In addition to the hot stamping process, which generates a full martensite phase, the soft phases are intended to form with reduced strength and higher ductility [3]. The mechanical properties of the B-pillar need to be “tailored”, that is, the bottom should have high energy absorption capability and the top should exhibit good intrusion resistance [4]. Maikranz-valentin et al. [5] proposed a hot stamping strategy using differential heating and cooling combined with thermal–mechanical integration processes for customized microstructure design, so as to obtain complex parts with expected performance. In order to tailor the mechanical properties of components, Liang et al. [6] used resistance heaters in the hot stamping tools, where the tools can be classified into heated zone, transition zone, and unheated zone. By controlling the heating and cooling rate in segmented tools, George et al. [7] obtained lab-scale B-pillars with customized performance.

Accurately predicting the mechanical properties of boron steel hot stamping is very important to study its formability. Many researchers have explored the microstructure evolution, mechanical characterization, and failure mode of boron steels. Cui et al. [8,9] proposed a numerical method to depict the effect of holding time on the mechanical properties and microscopic morphology of BR1500HS after hot stamping. In order to predict hardness distribution and microstructure evolution, Tang et al. [10] proposed a fully thermo–mechanical–metallurgical FE model, considering the influence of phase transformation kinetics in FE software Forge™ and verified it with laboratory-scale U-shaped channel parts. Bardelcik et al. [11] established a “Tailored Crash Model” as the constitutive model for as-quenched mixed B/M microstructures, considering the effect of strain, strain rate, and phase fractions. Bardelcik et al. [12] produced the as-formed microstructures including ferrite, bainite, and martensite in the miniature tensile specimens. They established a “Tailored Crash Model II” to consider the effect of deformation and microstructure in constitutive modeling. The model was verified to be capable of predicting the strain hardening performance of multiphase material under various strain rate ranges. T.K. Eller et al. [13] generated martensite, bainite, and ferritic/pearlite microstructures, and modeled their constitutive equations using an extended Swift hardening law and a stress state sensitive damage model. The behavior of the in-between hardness material grades can be estimated by piecewise linear interpolation of these three calibrated constitutive models for individual hardness grades. In order to study the influence of loading state on fracture initiation, Mohr et al. [14] studied the deformation behavior and fracture characteristics of martensite steel in plane stress loading. Tang et al. [15] proposed the extended DF2015 ductile fracture criterion to predict the failure behavior of quenched 22MnB5 sheet specimens under varying loading conditions. Wang et al. [16] chose Johnson-Cook (J-C) and Gurson-Tvergaard-Needleman (GTN) models to investigate the damage behavior of boron steel sheets with different hardness. Eller et al. [13] established a modified Mohr-Coulomb (MMC) fracture criterion for three hardness grades of 22MnB5 sheets. The results showed that the steels composed of ductile ferrite/pearlite and bainite phases had higher ductility than those containing brittle martensite phases.

The mechanical behavior and damage evolution of steels composed of multiphases has been described by micromechanical methods. Sodjit et al. [17] generated 2D RVE models to predict the overall strain-hardening of DP steels with different volume fractions of martensite. Based on dislocation theory and local chemical composition, the constitutive relations of individual martensite and ferrite phases were analytically formulated. Phetlam and Uthaisangsuk [18] proposed a stress–strain model for hardened steel JIS SNCM 439, considering the different hardening effects of individual constituents. A microscopic 2D RVE model was used to predict the macro constitutive relation of the steel under different heat-treatment conditions. Anbarlooie et al. [19] introduced 3D RVE models to study the material properties and deformation behavior of DP steel. The influence of volume fraction and element size on 3D simulations of overall strain-hardening was explored. Ramazani et al. [20] introduced 2D/3D correlation factors to transform the flow stress of DP steel obtained by 2D and 3D RVE methods. The fracture toughness of boron steel was also significantly affected by its multiphase microstructure. Golling et al. [21] used the essential fracture work method to predict the fracture behavior of boron steel commonly used in automobile manufacturing. In order to quantitatively evaluate the damage nucleation of DP steel under different stress states from micro and macro perspectives, Lian et al. [22] developed a micromechanical model. They found that the stress triaxiality and Lode angle have a significant influence on damage initiation.

In recent years, there have been extensive studies on the micromechanics and microstructural dependency induced by damage [23]. Hoefnagels et al. [24] studied the micromechanical effect of the observed damage mechanism in detail by in-situ scanning electron microscope tests, quantitative damage analysis, and a finite element simulation. According to mesoscopic damage mechanics, before ductile fracture the plastic deformation process includes void nucleation, growth, and coalescence. Void nucleation is particularly important since it is the signal of failure initiation. There are three damage initiation modes recognized in DP steels [25]: ferrite grain boundary (F/F) decohesion, ferrite/martensite (F/M) interface decohesion, and the martensite cracking. However, the dominant damage initiation mode depends on the microstructure of martensite, such as its hardness, size, distribution, and volume fraction. It is identified that the dominate damage initiation model converts from ferrite/martensite interface debonding to martensite cracking with a rising Vm [26]. Ismail K et al. [27] suggested that DP steels exhibiting platelet-like morphology had a good cracking resistance. When compared with blocky martensite, fibrous martensite is more prone to cracking [28]. Avramovic-Cingara G et al. [29] found that steel with uniformly distributed martensite showed a delayed void growth rate and continuous void nucleation during deformation. The stress/strain distribution characteristics between ferrite and martensite changed because of grain refinement, which improved martensitic toughness and interfacial cohesion [30]. Yan et al. [31] found that the void initiation of DP steel critically depended on the local martensite morphology or the local ferrite and martensite morphology. In addition, the dominant damage nucleation mode varied during the plastic deformation. Saeidi et al. [32] found that martensitic cracking occurred at lower plastic strain, while F/M debonding dominantly induced at higher strain before fracture. Tang et al. [25] studied the mesoscopic origin of damage nucleation in DP steel with different Vm through SEM, strain measurement, and numerical simulation. They found that the main mesoscopic causes of F/F debonding, F/M debonding, and martensitic cracking were sensitive to high plastic strain, plastic strain gradient, and stress, respectively.

There are considerable studies devoted to the elastoplastic models and damage criteria of DP steel sheet consisting ofthe mixed F/M [33,34], but few have addressed the void nucleation and growth of mixed B/M heterogeneous boron steel sheets with varying volume fractions. This work aimed to predict the constitutive equation and ductile damage evolution of quenchable boron steel 22MnB5 with varying volume fractions of martensite and bainite phases, and to consider its microstructure characteristics from the micro- to the macro-scale. Varying volume fractions of B/M mixture were produced by heat-treatment operations. Uniaxial tensile tests of dogbone specimens were implemented to obtain the constitutive equations of the heterogeneous sheet specimens. In addition, varying shaped samples for tensile testing were carried out using digital image correlation (DIC) techniques. The stress–strain relation obtained by dislocation theory and local chemical compounds was utilized to predict the constitutive equations for B/M mixtures. The damage curve of each phase was achieved by the hybrid experimental–numerical method, which was used as the ductile fracture criterion of each phase in RVE simulation, so as to ascertain the damage evolution of mixed B/M steel on the micro-scale. Finally, through SEM observations and finite element analysis, the void nucleation modes of two different B/M volume fractions were comparatively studied and experimentally validated.

## 2. Materials and Methods

### 2.1. Material Characterization

**Figure 1:**
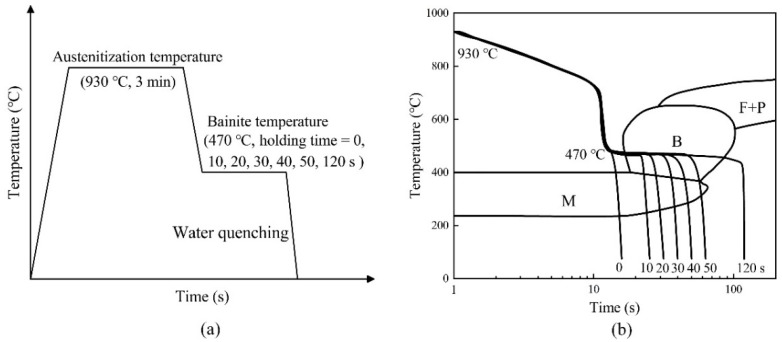
Heat treatment process in this study: (**a**) schematic diagram of heat-treatment procedures; (**b**) schematic diagram of time-temperature cycles with 22MnB5 CCT.

The material used is ultra-high strength boron steel 22MnB5 with an Al–Si coating, commercially known as Usibor 1500P. In the experiment, the thickness of the sheets selected is 1.8 mm. The measured chemical compositions of the selected 22MnB5 boron steel sheet are listed in Table 1. In order to study the deformation behavior in the tailor-distributed mechanical properties of hot stamping parts, bainite and martensite phases with different volume fractions were obtained by different heat-treatment processes. Seven microstructural features were prepared, including pure martensite, pure bainite, and mixed B/M heterogeneous products with five phase volume fractions. To begin with, 22MnB5 sheets were heated to the austenitization temperature of 930 °C, with a heating rate of 15 °C/s. After 3 min maintenance in a resistance furnace (Jinan Precision Scientific Instrument Co., Ltd, Jinan, China), the heated specimen was immediately transferred to a flat die where it was quenched to obtain pure martensite. Other specimens were moved to an electrically-heated flat die where they were cooled down to 470 °C, inducing bainite phase transformation. Different maintenances were applied to the specimens in the heated die, including 10, 20, 30, 40, 50, and 120 s [35]. The process flow of heat treatment is shown in the time–temperature cycle diagram in Figure 1a. The temperature change of heat-treated boron steel samples was measured by K-type thermocouples (Shanghai Automation Instrument Factory, Shanghai, China). Figure 1b shows the CCT diagram of 22MnB5 steel and the temperature change curves of the samples during the whole heat-treatment process. It was observed that all intermediate quenching paths were in the bainite phase transformation range, and the samples were maintained for varying holding times. Since the maintenance of 120 s is beyond the bainite phase transformation zone, the complete bainite microstructure is anticipated.

**Figure 2:**
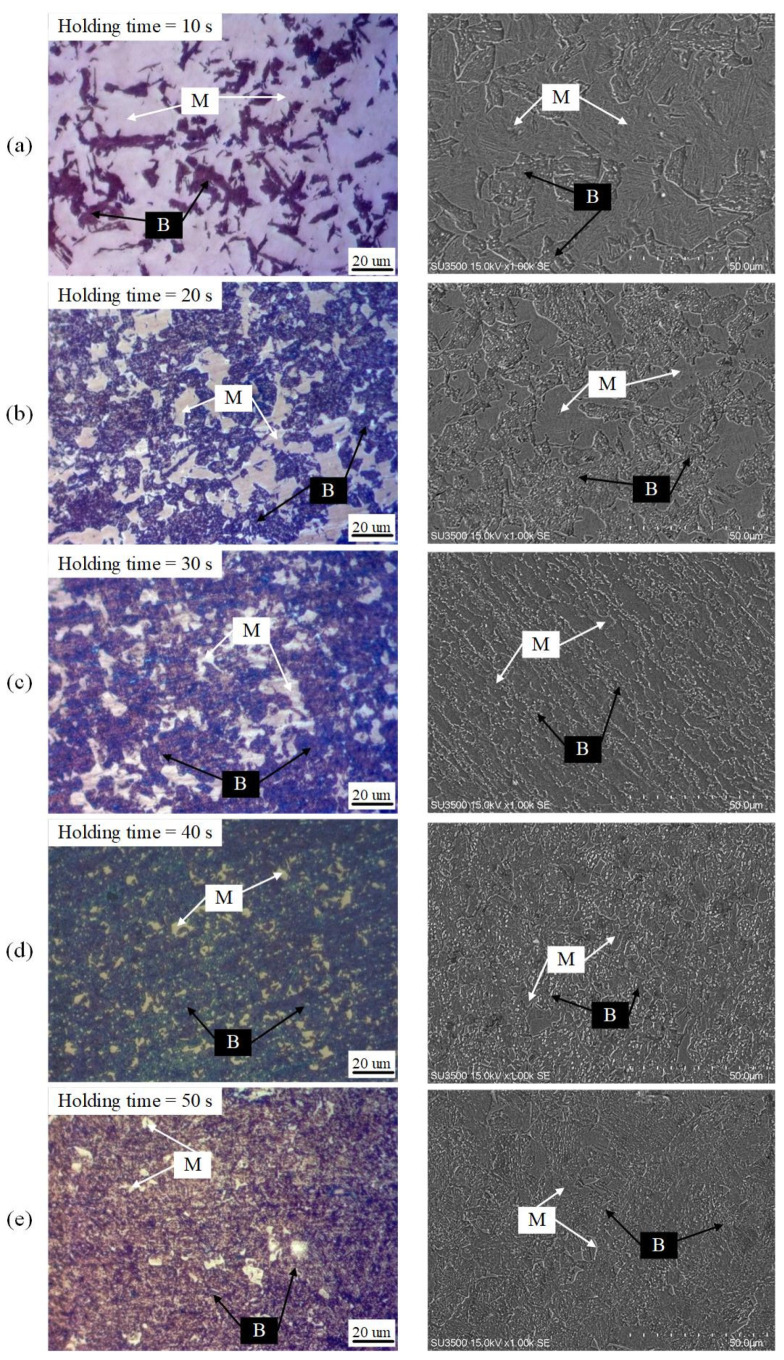
Optical micrographs (**left**) and SEM images (**right**) of heat-treated samples with varying holding times (**a**) 10 s, (**b**) 20 s, (**c**) 30 s, (**d**) 40 s, and (**e**) 50 s (In the figure, B and M represent bainite and martensite, respectively.).

The microstructural morphology was mainly composed of a mixture of bainite and martensite with different volume fractions under heat treatment. Note that the rolling direction of the sample is represented by the X axis, and the transverse and normal directions of the sample are represented by the Y and Z axes, respectively. The metallographic samples were cut from the Y–Z plane and then mechanically polished with sandpaper and a metallographic polishing machine (Jinan Fangyuan Testing Instrument Co., Ltd, Jinan, China) after inlaying. A two-stage color tint-etching approach (4% picral solution and 10% aqueous solution of sodium metabisulfite, that is, modified LePera etchant) was performed during metallographic experiments to identify as-formed microstructures and determine their volume fractions. Metallographic samples were characterized by a Leica DM2700M microscope (Leica Microsystems GmbH, Wetzlar, Germany). Figure 2 (left) shows the observed etching microstructure and multiphase. According to the degree of erosion, the shallow contrast display is marked as martensite and the dark contrast display is regarded as bainite. After re-grinding and polishing the metallographic specimens, it were etched with a 4% Nital solution. Scanning electron microscope experiments were carried out with 10 kV secondary electrons on an SU3500 instrument (Shimadzu (Shanghai) Global Laboratory Consumables Co., Ltd, Shanghai, China). The SEM images of the corresponding samples are depicted in Figure 2 (right). Bainite structure is a ferrite matrix with dispersed fine cementite particles. Optical micrographs and SEM images were automatically identified and divided into two distinct components by using Photoshop (Adobe Photoshop CC 2019) [36]. Segmentation was performed by adjusting contrast and color so that all martensite crystals were white and all bainite crystals were black. Then, the volume fraction of bainite and martensite was obtained by calculating the area of different colors. Table 2 shows that the volume fractions detected by the modified LePera etchant (Jinan Dongmu Biotechnology Co., LTD, Jinan, China) are similar to those of SEMs, which proves that the two-stage color tint-etching approach and SEM can well characterize the phase volume fractions of hot stamping boron steel 22MnB5. With the increase of holding time, the volume fraction of martensite decreases significantly.

### 2.2. Tensile Tests

#### 2.2.1. Dogbone Tensile Test

In order to characterize the constitutive models of martensite, bainite and mixed B/M phases, a series of dogbone tensile tests were carried out at a quasi-static strain rate. The dogbone specimens had a gauge length of 42 mm along with a nominal width of 10 mm. Before the experiment, specimen surfaces were polished to guarantee the adhesion of stochastic patterns (polishing softly with 600-grit abrasive paper). The white matte paint was sprayed on the surface of the specimen. While the paint was drying in the air for 3–5 min, black matte paint wss sprayed on the top layer of while paint, uniformly. The specimens after painting were advised to finish the test in one hour in case the priming paint fell off after drying. GOM ARAMIS^®^ AdjusTable 6M were used for measuring displacement and strain field by recording the deformation process with 10 images per second. Digital image resolution of 28 pixels per millimeter was set to the recording camera during tensile tests.

#### 2.2.2. Tensile Tests in Different Stress States

**Figure 3:**
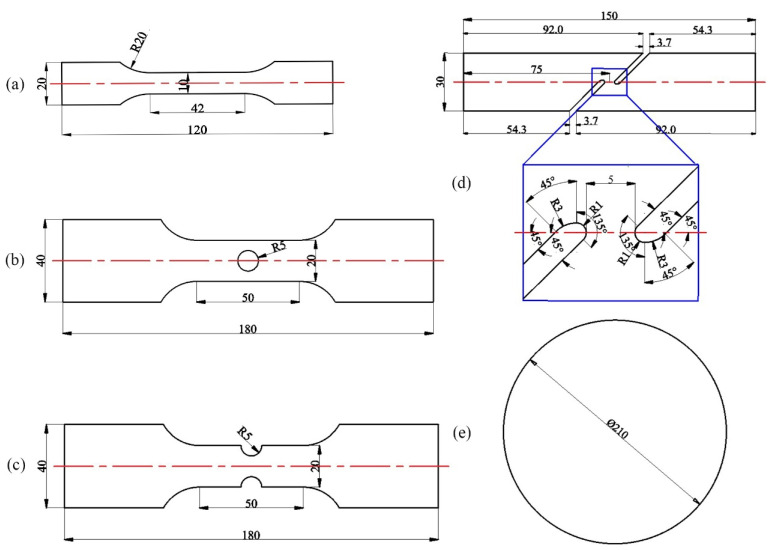
Geometrical dimension of five tensile specimens: (**a**) dogbone; (**b**) central hole; (**c**) notched (R5); (**d**) in-plane shear; and (**e**) Nakajima (Units: mm).

Notched, central hole, in-plane shear, and Nakajima specimens shown in Figure 3 were produced to characterize the evolution of ductile fraction of 22MnB5 sheets. The Nakajima tests were carried out on a universal sheet metal-testing machine Genbon EC600 (Shanghai Jinbang Industrial Co.,Ltd, Shanghai, China). All experiments were assumed to be carried out under quasi-static states without considering the influence of strain rate on ductile fracture mechanisms [37]. Table 3 shows the tensile speed of the specimens determined by finite element analysis to assure that the strain rate was about 0.001/s [38]. In case of the Nakajima test, a GOM ARAMIS 3D system was applied to record displacement and strain field at a rate of 5 per second, with a resolution of 15 pixels per millimeter at the fracture stroke.

## 3. Micro-Mechanical Modeling

### 3.1. Representative Volume Element (RVE) Approach

RVE analysis is a popular method for the statistical analysis of multiphase materials. Because the response of each phase affects the overall macro-mechanical behavior of the specimen, RVE shall be of suitable size and all relevant constituent phases must be included. The 2D RVEs were generated directly from the random area of the real light micrographs. The overall size of the RVE was confined to 80 µm × 80 µm. The selected square with heterogeneous microstructure was automatically identified and divided into two distinct components by using Photoshop [36]. Segmentation was performed by adjusting contrast and color so that all martensite crystals were white and all bainite crystals were black. Then, OOF2 software [39] was selected to transform the grayscale image into finite element mesh. The element size of the 2D RVE was 0.5 µm × 0.5 µm. Finally, the generated grid file was imported into ABAQUS for subsequent simulation calculation.

### 3.2. Constitutive Modeling

#### 3.2.1. Stress–Strain Curve

**Figure 4:**
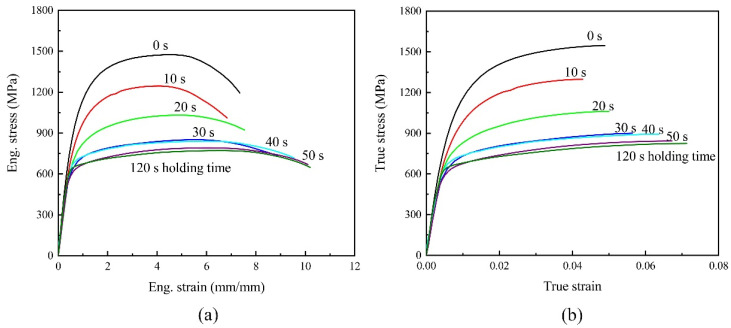
Flow curves of dogbone tensile specimens with different holding times: (**a**) engineering stress–strain curves; (**b**) true stress–strain curves.

According to the results of the dogbone tensile test, the averaged engineering and true stress–strain curves with varying holding times are respectively presented in Figure 4a,b. The specimens without holding exhibit the highest ultimate tensile strength (UTS) of 1500 MPa due to fully transformed martensite. Specimens maintained for bainite transformation have low strength due to the presence of mixed B/M microstructures with different volume fractions. It can be seen that in the range of bainite transformation temperature, the yield strength and tensile strength gradually decrease, with the increase of holding time. The elongation of the specimen increases significantly once the holding time is longer than 30 s, providing a high capability of energy absorption. Though bainite elongation is the highest, its ultimate tensile strength reduces to 770 MPa.

#### 3.2.2. Constitutive Modeling of Individual Phases

Material properties of bainite and martensite phases were assumed to be homogenous and isotropic [40]. It was assumed that both phases have Young’s moduli of 180 GPa and a Poisson’s ratio of 0.3. The constitutive relations of pure martensite and bainite phase were defined based on the dislocation strengthening theory, which was introduced by Rodriguez et al. [41,42]. Equation (1) denotes the relation of true stress and true plastic strain.

(1) $$\sigma =\sigma_{0}+\Delta \sigma +\alpha M\mu \sqrt{b}\sqrt{\;\frac{\;1-exp\;\left(-Mk\varepsilon \right)}{kL_{k}}}\;\;\;\;\;\;$$

The first term, $\sigma_{0}$, indicates the Peierls stress and it is the function of alloying elements and calculated by Equation (2). σ and ε denote true stress and true plastic strain, respectively. The second term, Δσ, in Equation (1) takes into account precipitation or solution strengthening, which is considered as a fitting parameter in the study. $L_{k}$ is the dislocation’s mean free path. k represents the rate of dynamic recovery. $L_{k}$ and k depict the effect of strengthening and softening, respectively. α denotes a material constant, μ represents the shear modulus. M and b is the Taylor factor and Burgers vector length, respectively.

(2) $$\;\sigma_{0}=77+80\left(\%\mathrm{Mn}\right)+750\left(\%P\right)+60\left(\%\mathrm{Si}\right)+80\left(\%\mathrm{Cu}\right)+45\left(\%\mathrm{Ni}\right)+60\left(\%\mathrm{Cr}\right)+11\left(\%\mathrm{Mo}\right){+5000(\%N}_{\mathrm{ss}})$$

**Figure 5:**
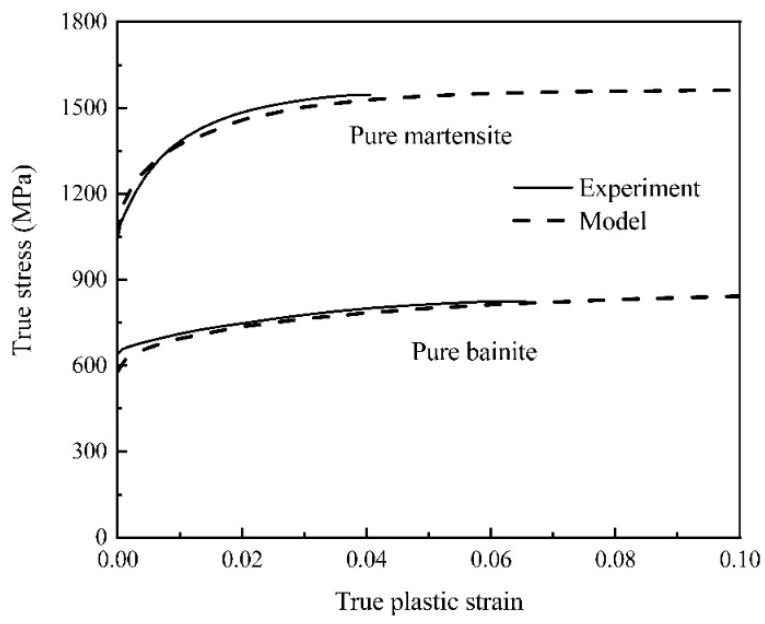
Flow curves for pure martensite and pure bainite specimens were simulated and compared with experimental data (solid lines represent experimental values and dash lines represent fitting values in constitutive model).

**Figure 6:**
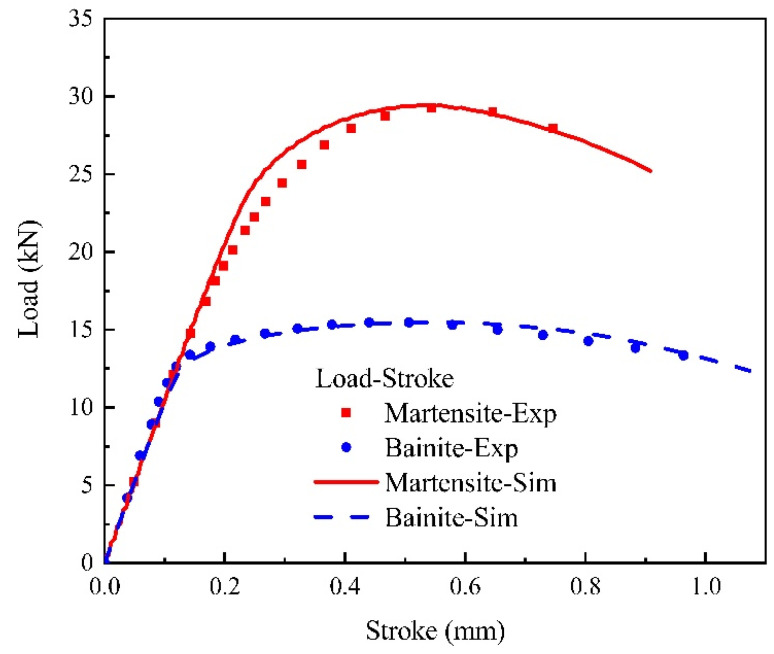
Load–stroke curves comparison for martensite and bainite specimens, respectively (experiment vs. simulation).

Material parameters in Equations (1) and (2) for martensite and bainite uniaxial tensile specimens were optimized until the predicted results meet the experimental data at a satisfactory level, as shown in Figure 5. The values of the optimized material parameters of the two phases are given in Table 4. In order to verify the predictability of the constitutive equations, notched specimens of bainite and martensite were stretched at a strain rate of 0.001 s^−1^. The load–stroke curves were predicted using stress–strain curves, optimized by the reverse engineering method [43], and the results were consistent with the experimental results, as shown in Figure 6.

#### 3.2.3. Constitutive Modeling of Mixed B/M Microstructure

During the bainite transformation process, the carbon content of the remaining austenite increases, leading to changes in the mechanical properties of the resulting martensite [44]. Therefore, the material parameters of martensite of different B/M phase fractions need to be calibrated. For martensite phase obtained with different holding time, it is considered that the change of mechanical properties is mainly induced by carbon content [3]. Therefore, in this study, the parameter Δσ was optimized, and the parameters $L_{k}$ and k were consistent with the pure martensite phase. Although not as obvious as martensite, the strength and hardness of bainite also show a correlation with carbon content. In Ref. [3], when compared with the specimens with other bainite volume fractions, the specimens with low bainite volume fraction show a higher work hardening rate, which may be due to the finer lath size of the low bainite structure. Thus, the bainite parameters of Δσ, $L_{k}$, and k are regarded as optimization parameters.

**Figure 7:**
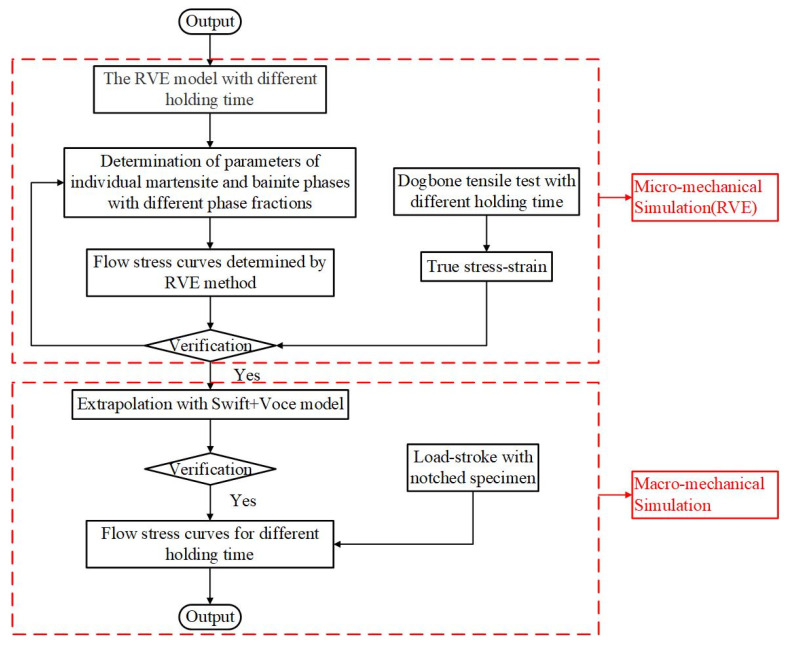
Flowchart to determine the stress–strain curves of bainite and martensite constituent phases.

Firstly, RVE models were established according to the observed microstructure with holding time of 10, 20, 30, 40, and 50 s (the corresponding B/M phase fractions of 30:70, 68:32, 80:20, 90:10, and 96:4, respectively). Based on the material parameters of bainite and martensite determined in Section 3.2.2, preliminary flow curves of tensile specimens with varying B/M phase volume fractions were defined. The flow stress–strain models of each phase were applied to the RVEs, and the output overall strain-hardening curves were compared with the experimental data. If the fitting was not satisfied, the material parameters of bainite and martensite phases would be updated. In this study, the mixed Swift-Voce strain hardening law [45] was chosen to determine the flow stress under large plastic strain. The true stress–true strain curves represented by the mixed constitutive model were applied to simulate the tension of notched specimens, and the load–stroke curves that were predicted were compared with the experimental results for the validation of the constitutive model. The flowchart to determine the stress–strain curves of bainite and martensite constituent phases is schematically summarized in Figure 7.

**Figure 8:**
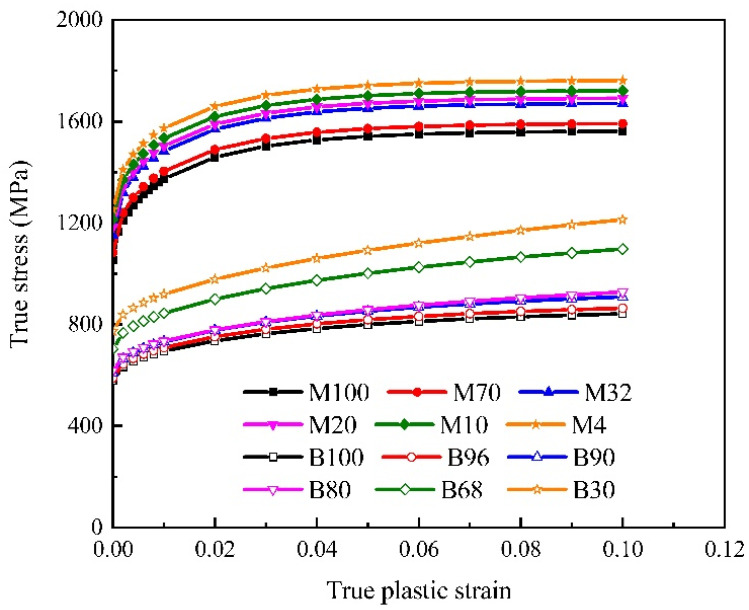
Flow curve predictions of the specimens with varying B/M phase volume fractions.

The determined flow curves of specimens with varying B/M phase volume fractions are shown in Figure 8. It can be found the flow curve of the specimens with 4% Vm are the highest among those of the specimens with 10, 20, 32, 70, and 100% Vm, due to its higher carbon content of the specimen with lower Vm [3].

**Figure 9:**
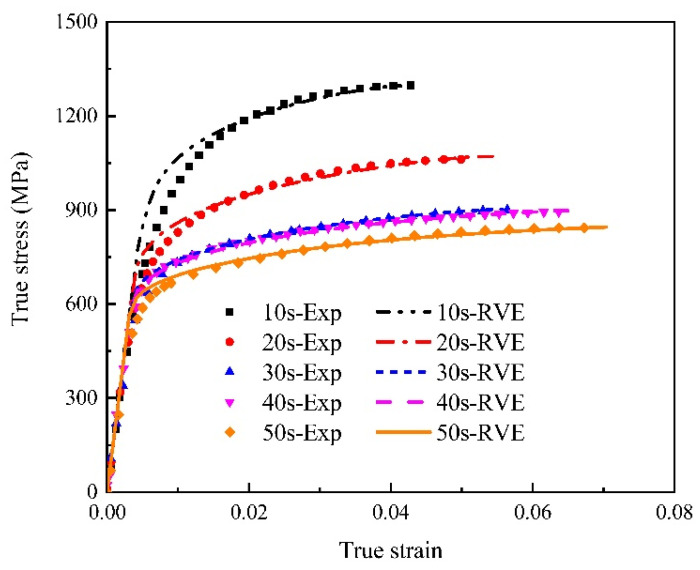
Comparisons of flow curves determined by RVEs with experimental results (the dots represent experimental values and the lines represent simulated values in RVE).

The bainite flow curves show identical characteristics, but with significantly lower flow stresses. The stress–strain response of martensite exhibits a higher strain hardening rate at the start of material flow and quickly reaches saturation flow stress at true plastic strain of around 0.06. The flow stress of the bainite flow curve increases continuously until reaching a high strain value. The bainite and martensite flow curves of specimens with varying B/M phase volume fractions were applied to the RVE model and the effective true stress–true strain curves were calculated as exhibited in Figure 9. The flow curves of specimens with different B/M phase fractions predicted by the RVEs are consistent with the experimental results. The constitutive parameters of bainite and martensite phases were optimized as shown in Table 5 and Table 6, respectively. These identified parameters were used for the following RVE simulations.

In the case of macroscopic FE simulation, the flow stress–strain curves obtained by experiments were extrapolated using the mixed Swift-Voce strain hardening law, which is expressed as follows in Equation (3).

(3) $$\sigma =\beta {(m(\varepsilon }_{0}{+\varepsilon )}^{n})+(1-\beta )(A-(A-B)\cdot \exp(-C\cdot \varepsilon ))$$

where σ and ε denote the true stress and true strain, respectively. There were seven parameters, β, m, $\varepsilon_{0}$, n, A, B, and C, to be calibrated and optimized.

**Figure 10:**
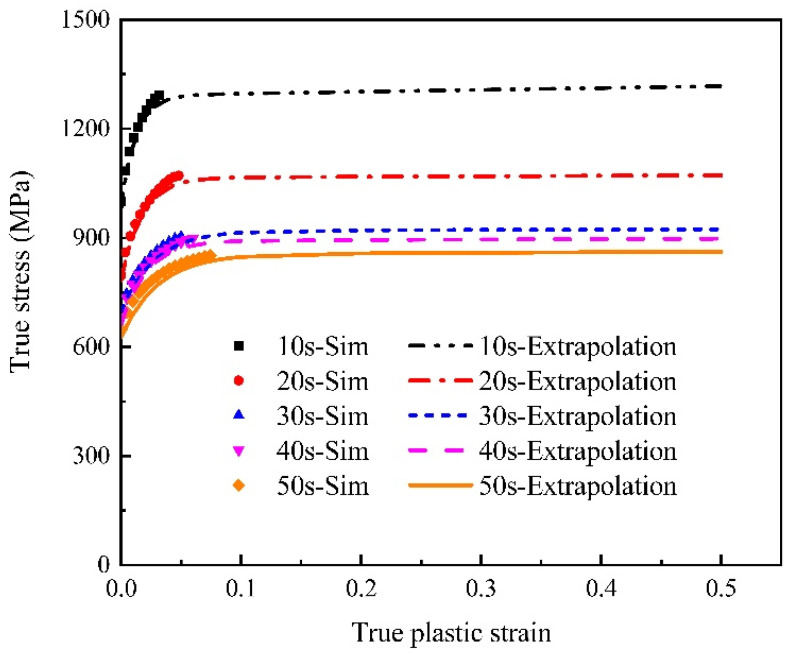
Flow stress curves of extrapolation for different holding times (the dots represent simulated values in RVE and the lines represent extrapolation values).

**Figure 11:**
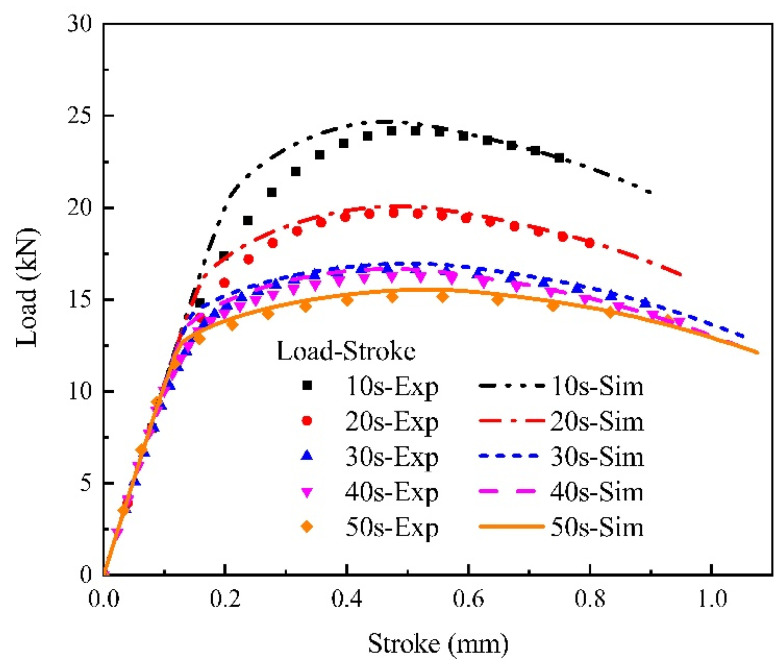
Load–stroke curve comparisons between experiment and simulation for different holding times (the dots represent experimental values and the lines represent simulated values).

Figure 10 illustrates the stress–strain curves at large plastic strain using the mixed Swift-Voce strain hardening law which was optimized by the reverse engineering method. As illustrated in Figure 11, the load–stroke curves obtained by the optimized hardening model are consistent with the experiments. The stress–strain curves was used in macro-mechanical simulations to obtain fracture strains.

#### 3.2.4. RVE Simulations for Uniaxial Tensile Specimens

**Figure 12:**
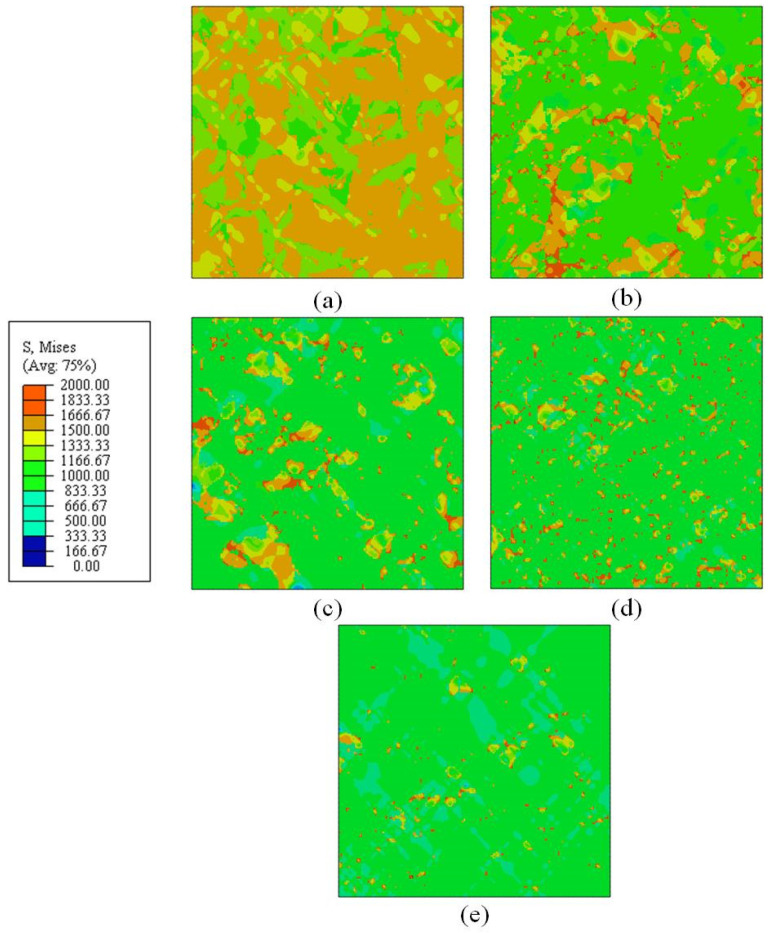
Predicted local distributions of von Mises equivalent stress at the overall strain of 7% for RVEs with varying B/M phase volume fractions (**a**) 30%B + 70%M; (**b**) 68%B + 32%M; (**c**) 80%B + 20%M; (**d**) 90%B + 10%M; (**e**) 96%B + 4%M (Units: Pa).

**Figure 13:**
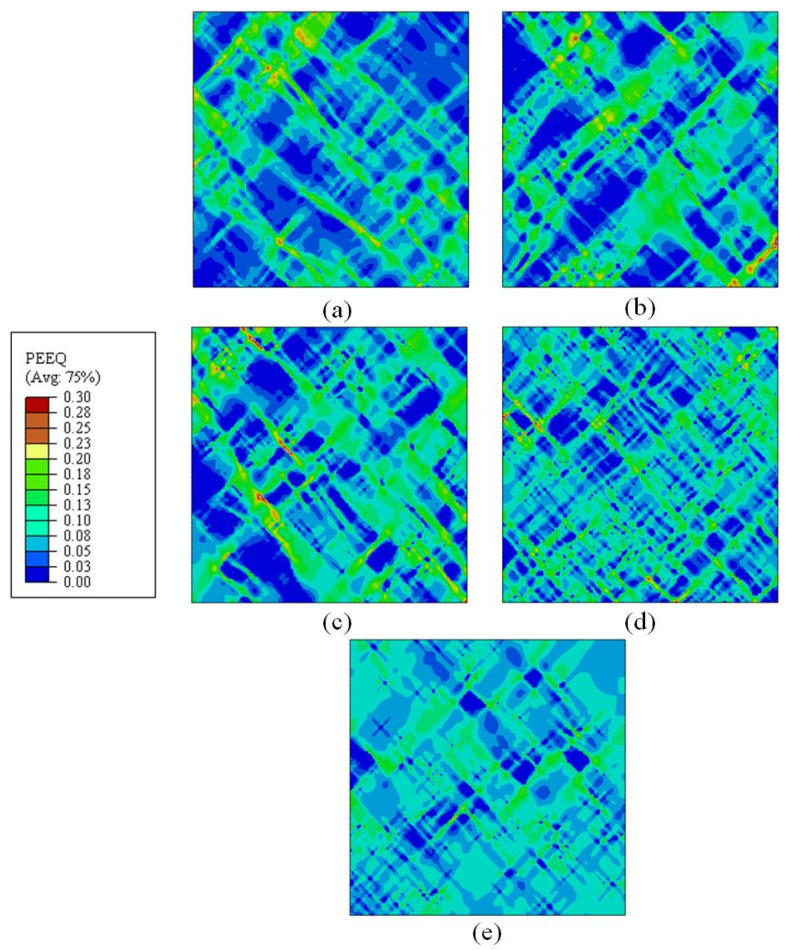
Predicted local distributions of equivalent plastic strain (PEEQ) at the overall strain of 7% for RVEs with varying B/M phase volume fractions (**a**) 30%B + 70%M; (**b**) 68%B + 32%M; (**c**) 80%B + 20%M; (**d**) 90%B + 10%M; (**e**) 96%B + 4%M.

Dual-phase 2D RVE models were used to predict the stress–strain relation of mixed B/M microstructures. The RVE models were generated based on the transformed microstructures with varying holding times of 10, 20, 30, 40, and 50 s. In order to study the distribution of plastic strain and von Mises equivalent stress, 2D plane strain (CPE4) elements were chosen to discretize the RVE FE models [3]. In the case of uniaxial stretching, nodes along the left edge of the RVE model were fixed in the X-direction but free in the Y-direction. Similarly, nodes along the bottom edge were fixed in the Y-direction but free in the X-direction. All nodes along the right edge of the RVE model had the same displacement in the X direction, while no constraints were imposed in the Y direction. The localized stress and strain distributions were predicted by the RVE simulations of uniaxial tension. The local distributions of von Mises equivalent stress and strain at an overall strain of 7% were obtained for RVEs of specimens with holding times of 10, 20, 30, 40, and 50 s, corresponding to B/M phase volume fractions of 30:70, 68:32, 80:20, 90:10, and 96:4, as displayed in Figure 12. It can be found that the harder martensite phase has an obviously better capability to carry stress than the softer bainite phase. This is due to the fact that the stress–strain distribution is dependent to a large extent on the morphology and distribution of the microstructure. At a holding time of 50 s (96%B + 4%M), the RVE with low martensite volume fraction shows the highest local stresses, which coincides with the trend of the stress–strain curve exhibited in Figure 8. Additionally, as shown in Figure 13, higher volume fractions of martensite in the B/M microstructure lead to significant local strain localizations for the RVE simulations with B/M volume fraction of 30:70 (30%B + 70%M), where distinctly and continuously distributed shear zones appear. In such microstructures with a high percentage of martensite matrix and a small amount of bainite phase dispersion, this can lead to earlier initiation of local damage. As shown in Figure 4, the elongation of the specimens with 30%B + 70%M is even a bit lower than that of the pure martensite specimens. Besides, the RVEs with a lower martensite volume fraction, as in the case of 90%B + 10%M shown in Figure 13, have a significantly more uniformly distributed plastic strain. These conclusions are consistent with the elongation of the tensile specimens determined in Figure 4.

### 3.3. Damage Prediction

#### 3.3.1. Damage Prediction of the Single Phase

Microscopically, metal plastic deformation involves void initiation, growth and coalescence [46,47]. In this paper, Lou-Huh damage criterion (DF2015) [47] was embedded into the simulation of the martensite and bainite tensile tests, respectively, which captures the influence of stress states on micro-scale. The Lou-Huh damage criterion is described in Equation (4).

(4) $${\left(\frac{2}{\sqrt{L^{2}+3}}\right)}^{C_{1}}{\left(\langle \frac{\eta +C_{4}\frac{\left(3-L\right)}{3\sqrt{L^{2}+3}}+C_{\text{offest}}}{\frac{1}{3}+\frac{2}{3}C_{4}+C_{\text{offest}}}\rangle \right)}^{C_{2}}{\overset{ˉ}{\varepsilon }}_{f}^{p}=C_{3}\;\;\langle x\rangle =\{\begin{array}{c} x\;when\;x\geq 0\\ 0\;when\;x<0 \end{array}$$

where ${\overset{ˉ}{\varepsilon }}_{f}^{p}$ denotes PEEQ to fracture, *η* and *L* are the stress triaxiality and the Lode angle parameter, respectively. $C_{1}{,\;C}_{2}{,\;C}_{3}{,\;C}_{4}$, and $C_{\mathrm{offset}}$ are fracture parameters that were required to be calibrated by experimental data.

In case of non-proportional loading, in order to consider the influence of the deformation evolution in complex sheet forming simulations, Equation (4) can be converted into its integral form. However, this paper focused on the study of tension from shear to plane strain and did not study the non-proportional strain loading

(5) $$\frac{1}{C_{3}}\int_{0}^{{\overset{ˉ}{\varepsilon }}_{f}^{p}}{\left(\frac{2}{\sqrt{L^{2}+3}}\right)}^{C_{1}}{\left(\langle \frac{\eta +C_{4}\frac{\left(3-L\right)}{3\sqrt{L^{2}+3}}+C_{offset}}{\frac{1}{3}+\frac{2}{3}C_{4}+C_{offset}}\rangle \right)}^{C_{2}}d\overset{ˉ}{\varepsilon }=D(\overset{ˉ}{\varepsilon })\;\langle x\rangle =\left\{ \begin{array}{l} x\;\text{when}\;x\geq 0\\ 0\;\text{when}\;x<0 \end{array}\right.$$

When the values of *D* and $D(\overset{ˉ}{\varepsilon })$ reach unity, the onset of fracture initiates. The determination of fracture strain is an enormous challenge in sheet metal stamping [47]. When specimens are subjected primarily to tensile stresses, the increase of void size due to the maximum value of stress triaxiality in the core provokes the initiation of ductile fracture. Since the fracture initiation in a material is usually located in the center of the sheet, it is impossible to accurately measure the true fracture strain using the DIC method. It was reported that the fracture strain at the center of the specimen was usually greater than the PEEQ of the corresponding specimen surface as measured by DIC [46].

**Figure 14:**
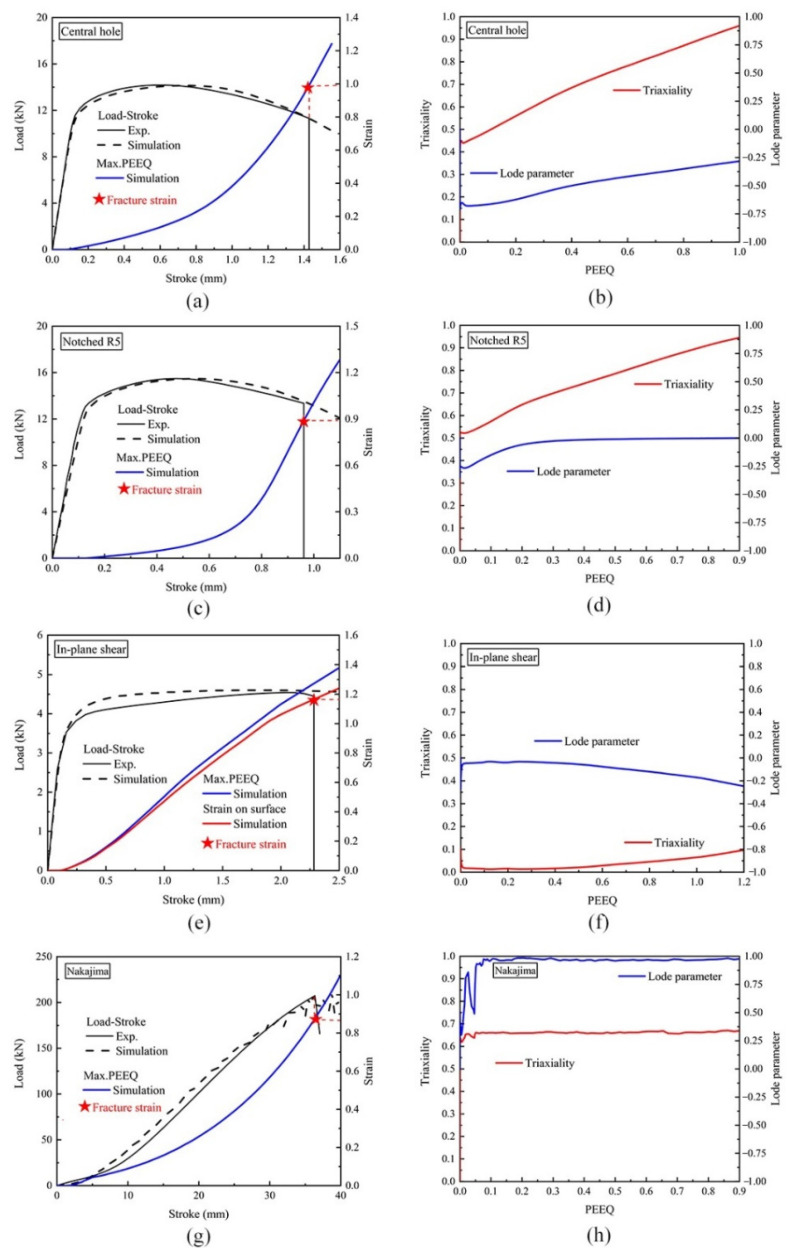
Numerical and experimental tensile results for different bainite specimens: (**a**) central hole, (**c**) notched, (**e**) in-plane shear, and (**g**) Nakajima specimen simulated load–stroke curves and PEEQ distribution, compared with experimental results; (**b**) central hole, (**d**) notched, (**f**) in-plane shear, and (**h**) Nakajima specimen calculated evolutions of the Lode parameter and the triaxiality towards PEEQ.

**Figure A1:**
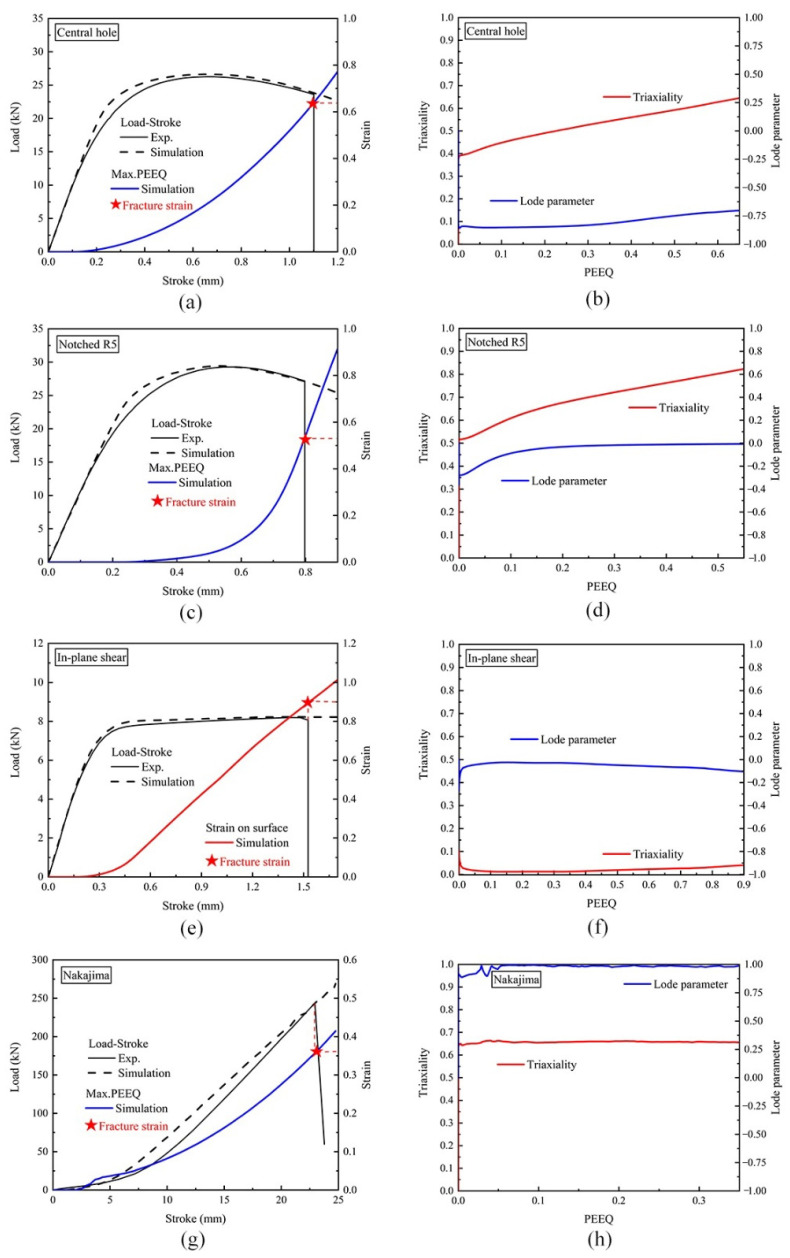
FE simulation results of tensile process for different martensite specimens: (**a**) central hole, (**c**) notched, (**e**) in-plane shear, and (**g**) Nakajima specimen simulated load–stroke curves and PEEQ distribution, compared with experimental results; (**b**) central hole, (**d**) notched, (**f**) in-plane shear, and (**h**) Nakajima specimen calculated evolutions of the Lode parameter and the triaxiality towards PEEQ.

The hybrid experimental–numerical approach proposed by Dunand and Mohr [48] was used in the paper to calculate the ductile fracture strain. Damage curves of the bainite and martensite phases were preliminarily calculated by the hybrid experimental–numerical approach. The FE simulations using the constitutive equations in Section 3.2.2 were used to predict the deformation process. Hexahedral solid elements with reduced integration (C3D8R) were chosen to discretize the FE model of specimens. In order to avoid shear locking of full numerical integration, the reduced numerical integration was used. Geometric symmetry as well as boundary condition symmetry was utilized to improve calculation efficiency. One-eighth of the finite element model was used for the central hole specimens and notched specimens. In the case of an in-plane shear specimen, only half of the specimen in the thickness direction was modeled in the FE simulation. Only the Nakajima specimen was modeled completely in the FE simulation [15]. During the simulation, the maximum PEEQ calculated before ductile fracture was defined as the ductile fracture strain. Figure 14 shows the comparisons of strain evolutions and load–stroke curves of bainite between simulations and experiments for central hole, notched, in-plane shear, and Nakajima specimens, respectively. The detailed process of fracture strain measurements is described in Ref. [15]. The comparison results of martensite can be referred to Figure A1 in Appendix A. The Lode parameter, stress triaxiality, and ductile fracture strain of bainite and martensite are recorded in Table 7 and Table 8, respectively.

**Figure 15:**
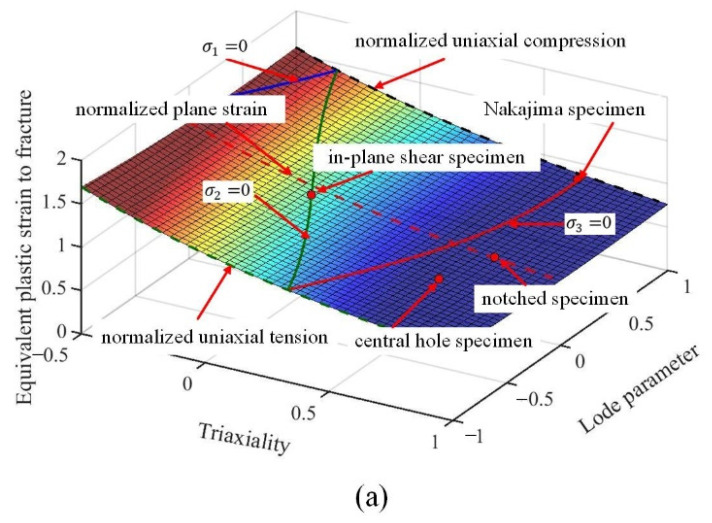

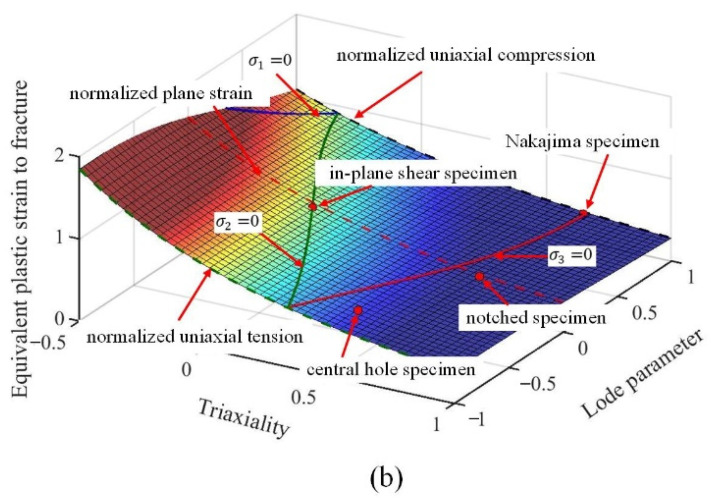
The constructed fracture loci optimized according to the predicted fracture strain in varying stress states and compared with experimental data: (**a**) bainite; (**b**) martensite.

Based on the experimental data summarized in Table 7 and Table 8, the fracture parameters of the Lou-Huh ductile fracture model were obtained by a simple linear regression, using least squares optimization [15]. The fracture loci of the individual bainite and martensite phase are depicted in Figure 15a,b, respectively. The fracture parameters of the pure bainite were optimized as $C_{1}$ = 0.3438, $C_{2}$ = 0.3938, $C_{3}$ = 0.0609, $C_{4}$ = 0.3411, $C_{\mathrm{offset}}$ = 0.8078; the pure martensite were optimized as $C_{1}$ = −0.1010, $C_{2}$ = 0.3786, $C_{3}$ = 0.7163, $C_{4}$ = 1.3105, $C_{\mathrm{offset}}$ = 0.9389. The constructed fracture loci meet most of the experimental fracture strains shown in Table 7 and Table 8 for both martensite and bainite specimens, which were incorporated into RVE simulations.

#### 3.3.2. RVE Simulations in Varying Stress States

The purpose of the section is to predict the damage initiation of B/M boron steels by RVE-based multiscale simulations under varying stress states. In ABAQUS, the plastic constitutive equation and damage defined curves of the individual phases are users’ subroutine VUMAT. For tailor-tempered 22MnB5 steels, ductile damage was induced by the incompatibility of deformation between martensite and bainite [35]. The B/M microstructure, consisting of 80% bainite and 20% martensite (80%B + 20%M), was selected for the investigation. The plastic constitutive equation and damage parameters of each phase were defined in the RVEs. The von Mises yield criterion and the associated flow rule, together with the mixed Swift-Voce strain hardening law was implemented to predict the behavior of non-linearly hardened materials.

**Figure 16:**
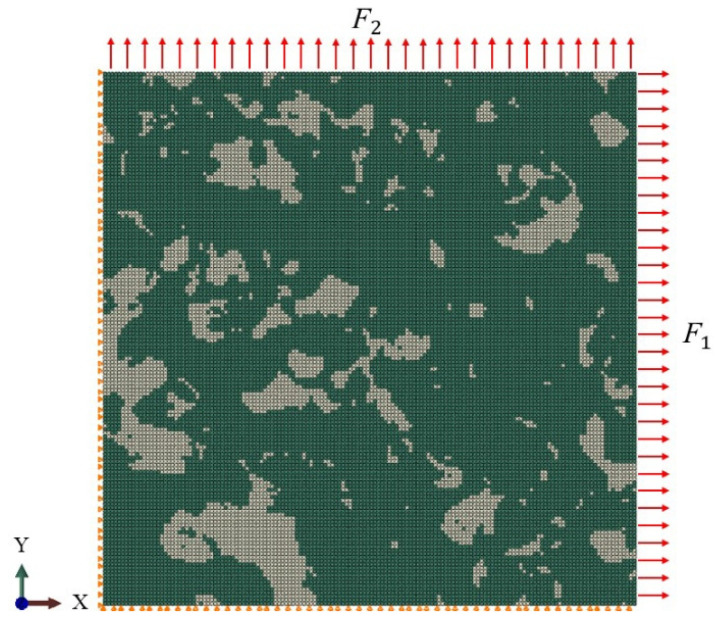
Boundary conditions for RVE simulations (the left edge and bottom edge were both fixed in the X- and Y-direction, and the uniform loads $F_{1}$ and $F_{2}$ were applied on the right and at the top which $F_{1}$ was fixed and $F_{2}$ was varied).

Four-node bilinear elements with reduced integration (CPS4R) were employed in the FE models. All the nodes along the left edge of the RVE were fixed in the X-direction, but free in the Y-direction, while the nodes along the bottom edge were fixed in the Y-direction, but free in the X-direction. The uniform loads $F_{1}$ and $F_{2}$ applied to all nodes on the right and at the top of the RVE, respectively, bring about varying stress states, as shown in Figure 16. Different values of overall stress triaxiality of the RVEs were induced by variations in the loads $F_{1}$ and $F_{2}$. In the study, $F_{1}$ was supposed to be constant, while $F_{2}$ was varied, as shown in Table 9, along with the corresponding stress triaxiality values. Since the distribution of the stress triaxiality in the model was not uniform, it was averaged in critical areas rather than the whole RVE region.

**Figure 17:**
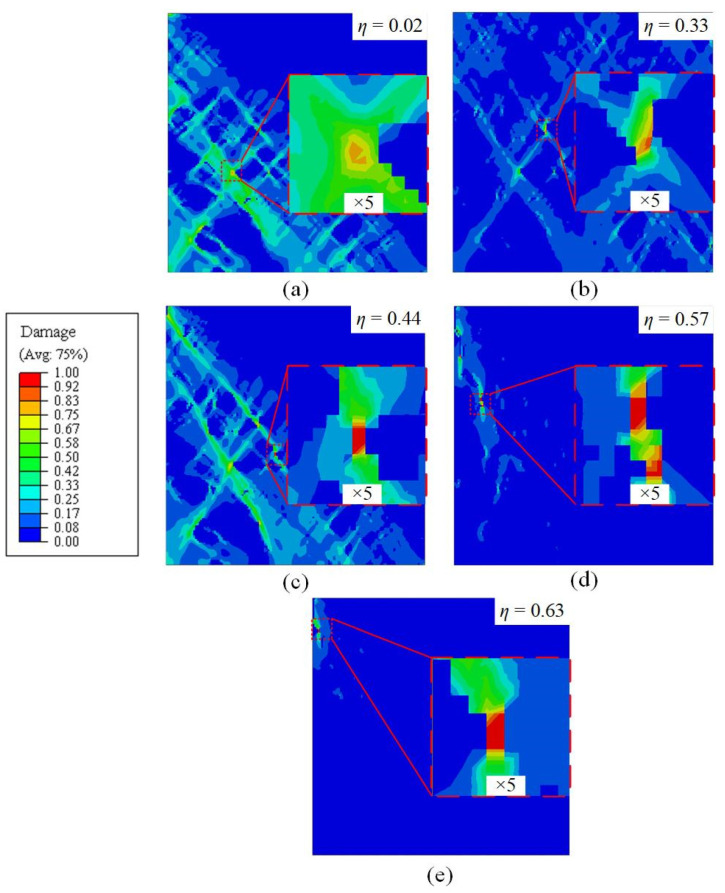
Simulated distribution of ductile damage initiation in RVEs for the mixed B/M microstructures in varying loading conditions: (**a**) pure shear; (**b**) uniaxial tension; (**c**) uniaxial tension with a central hole; (**d**) plane strain tension; (**e**) equi-biaxial tension.

During the numerical simulation of RVEs, the onset of damage is determined by the value of variable $D\left(\bar{\varepsilon }\right)$, as shown in Equation (5). If the value of $D\left(\bar{\varepsilon }\right)$ exceeds unity, damage is provoked and the element is removed. RVE simulations were carried out for mixed microstructure of 80%B + 20%M under different loading conditions. The distribution of ductile damage initiation values for the five stress states was predicted through the simulations and depicted in Figure 17. It can be observed that the occurrence of ductile damage is heterogeneous and different in the microstructure for different stress states. Red square dotted boxes in the RVEs are used to indicate the local regions where damage initiation occurred. Most of the damage initiation sites are located on shear bands and at the interface between bainite and martensite, particularly in the sharp edges and narrow crossover regions between the two phases, which will be discussed in Section 4. The PEEQs before the failure of the element are regarded as fracture strains for the mixed B/M microstructures in varying stress states.

#### 3.3.3. Damage Prediction of B/M Microstructures

**Figure 18:**
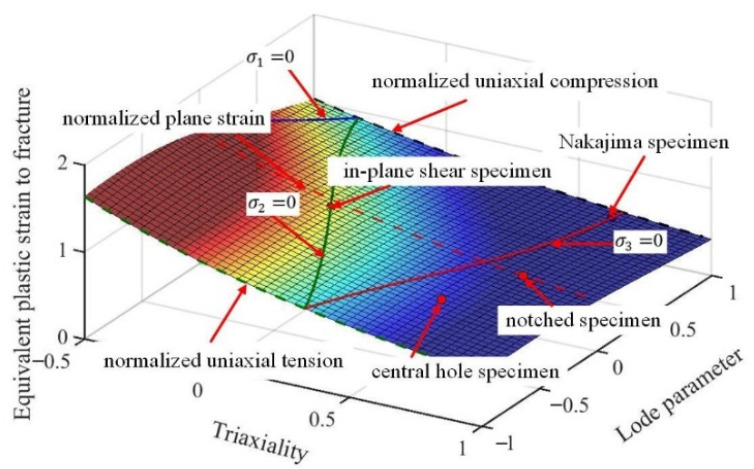
Fracture locus constructed based on the obtained fracture strains in different stress states and compared with experimental data for 80%B + 20%M specimens (red dots denote experimental values).

**Figure A2:**
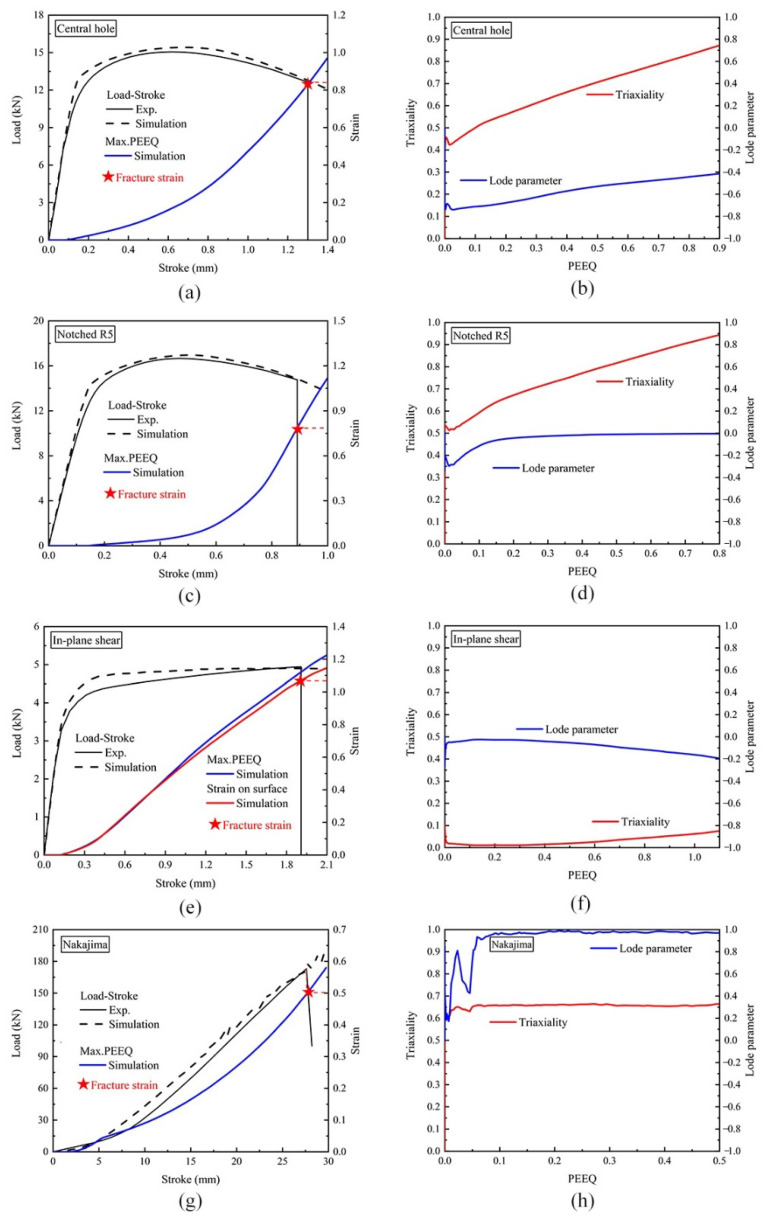
FE simulation results of tensile process for 80%B + 20%M specimens: (**a**) central hole, (**c**) notched, (**e**) in-plane shear, and (**g**) Nakajima specimen simulated load–stroke curves and PEEQ distribution, compared with experimental results; (**b**) central hole, (**d**) notched, (**f**) in-plane shear, and (**h**) Nakajima specimen calculated evolutions of the Lode parameter and the triaxiality towards PEEQ.

For the determination of damage curve of mixed B/M microstructure (80%B + 20%M), the fracture strains for varying stress states were firstly determined in the macroscopic scale by the hybrid numerical–experimental approach. FE simulation results of 80%B + 20%M specimens under varying loading conditions are shown in Figure A2 in Appendix A, including the evolutions of load, PEEQ, the Lode parameter, and the stress triaxiality. Fracture stains, the Lode parameter, and the stress triaxiality obtained by hybrid numerical–experimental approach for 80%B + 20%M specimens are shown in Table 10. The fracture parameters are optimized as $C_{1}$ = −0.3821, $C_{2}$ = 3.9011, $C_{3}$ = 0.9082, $C_{4}$ = −1.9446, $C_{\mathrm{offset}}$ = 6.9002. The fracture locus of 80%B + 20%M dual-phase microstructure is constructed in Figure 18.

**Figure 19:**
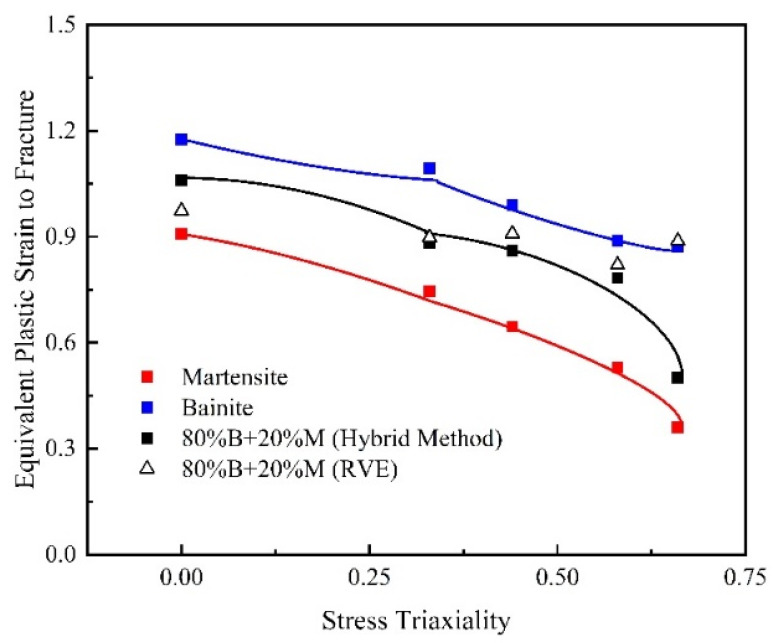
Predicted damage curves for B/M mixed microstructure and compared with damage curves of pure bainite and martensite (blue indicates bainite specimens, red indicates martensite specimens, and black indicates 80%B + 20%M specimens).

Damage curves for the B/M microstructures were constructed using the damage strains and the averaged stress triaxialities calculated by the RVEs. As exhibited in Figure 19, the predicted damage curves by RVE method are plotted, including the damage curves of pure bainite, pure martensite, and the mixed 80%B + 20%M microstructure calculated by the hybrid numerical–experimental method. The damage curve of the 80%B + 20%M mixed microstructure is situated between those of the pure bainite and pure martensite microstructure.

## 4. Results and Discussion

**Figure 20:**
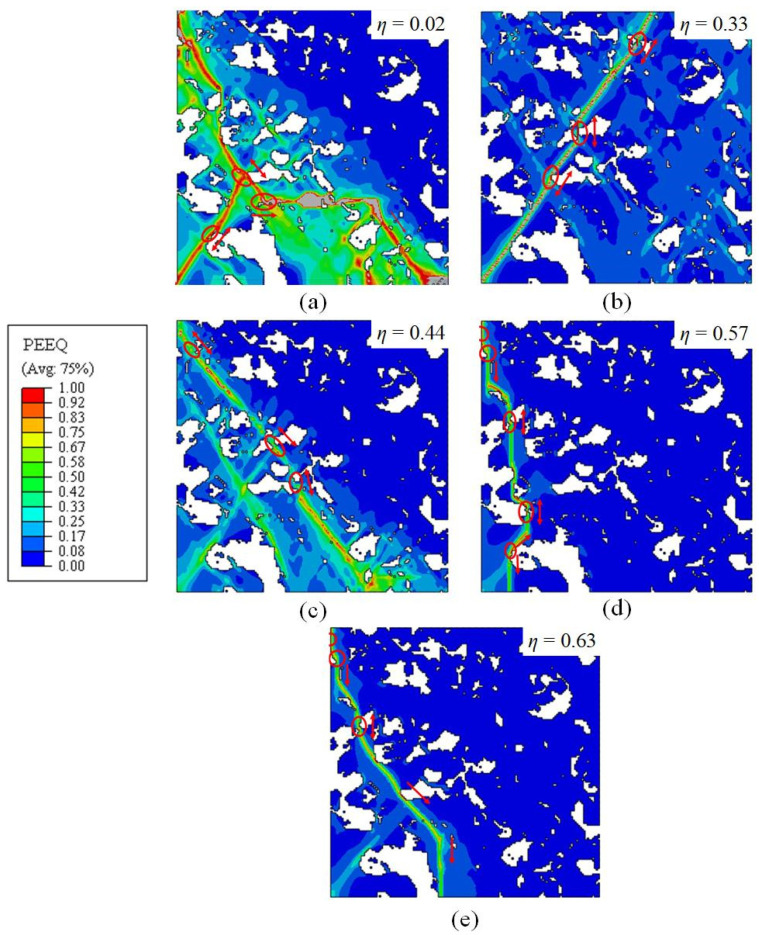
Calculated PEEQ in RVE under different loading conditions for the B/M microstructure (80%B + 20%M): (**a**) pure shear; (**b**) uniaxial tension; (**c**) uniaxial tension with a central hole; (**d**) plane strain tension; (**e**) equi-biaxial tension (white zones indicate the martensite phase, red circles denote the initiation positions of the failure and arrows indicate the directions of SBP).

In order to study the damage mechanism of B/M microstructure, SEM observations and FE simulations were carried out. Figure 20 shows the PEEQ distributions in 2D RVE models of the 80%B + 20%M microstructure under different loading conditions. The failure initiation positions are denoted by red circles and the directions of the shear band propagation (SBP) are indicated by red arrows. Five different damage scenarios are demonstrated in the RVE models with identical microstructure morphology. It was found that the loading conditions have a significant influence on the damage scenarios on the microscopic scale. This is because the stress states of the critical zones are divergent under all loading conditions, which are shown in Table 9. Consequently, under different loading conditions, the damage initiation sites and the failure evolution paths are different. Under the shear stress state (η=0.02), two main shear bands are formed in Figure 20a, and were found to intersect at the fracture stage. The failure in localized shear bands inclines to the direction of stretching, as displayed in the Figure 20b–d. A large number of shear bands would be formed under low stress triaxialities. In case of plane strain (η=0.75), the shear bands are fewer and propagate vertically to the loading direction of $F_{2}$. Due to the heterogeneity of the microstructure, the local PEEQ in bainite is concentrated near the B/M boundary for all five stress states. The simulated PEEQ distributions verify that fracture is initiated in bainite at all stress states, as shown in Figure 20. The microstructure inhomogeneity results in the starting positions of the failure adjacent to the B/M boundary, but the initiation locations are different for different stress states. Stress triaxiality plays a significant role in ductile failure initiation. At low stress triaxialities, failure tends to initiate at the B/M boundary, while at intermediate and high stress triaxialities, failure is prone to initiate in the bainite island surrounded by martensite phases. It is proved that the fracture initiation of B/M mixed microstructure is sensitive to both the microstructure and macroscopically applied stress state.

**Figure 21:**
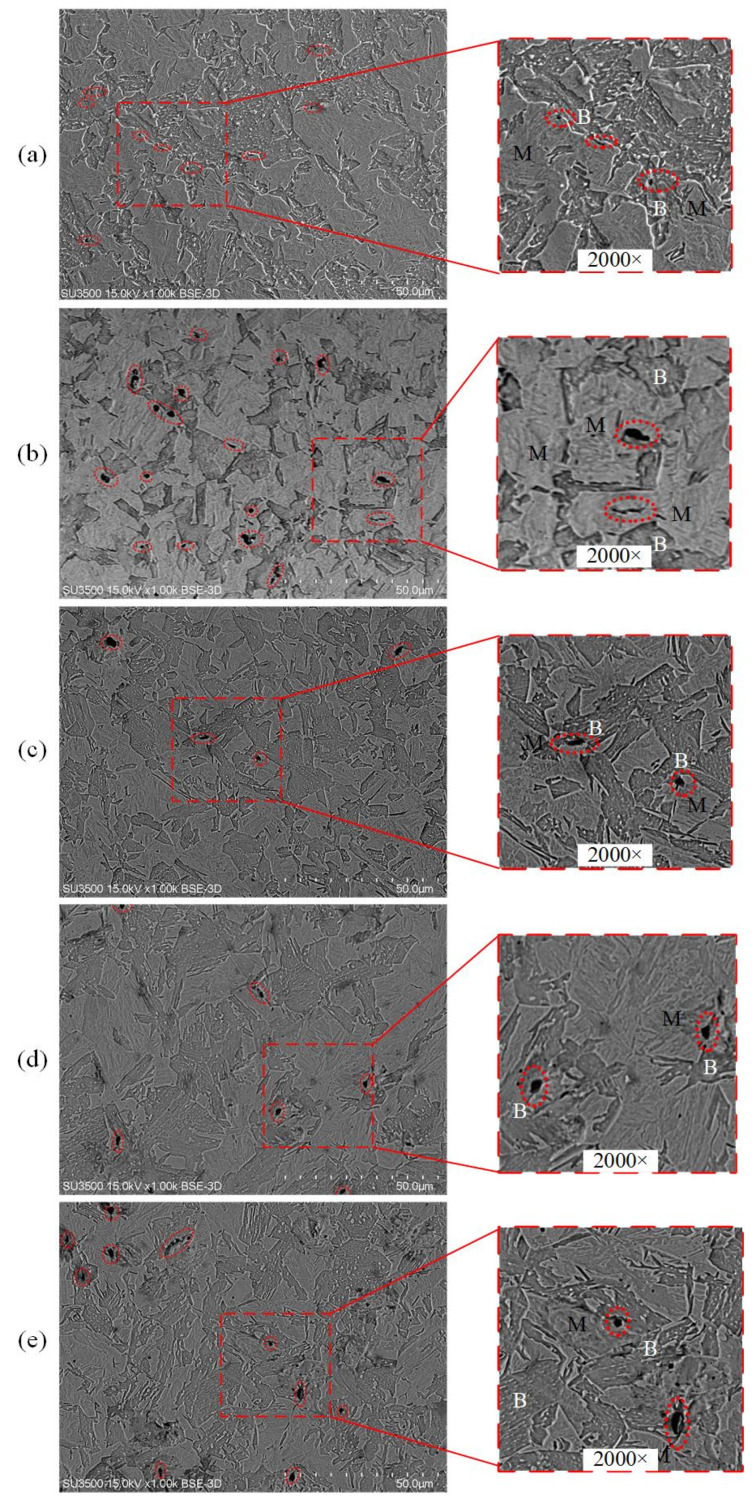
SEM micrographs showing void initiation and crack growth for 30%B + 70%M specimens under varying stress states: (**a**) pure shear specimen; (**b**) dogbone uniaxial tension specimen; (**c**) uniaxial tension specimen with a central hole; (**d**) plane strain tension specimen; (**e**) equi-biaxial tension specimen.

**Figure 22:**
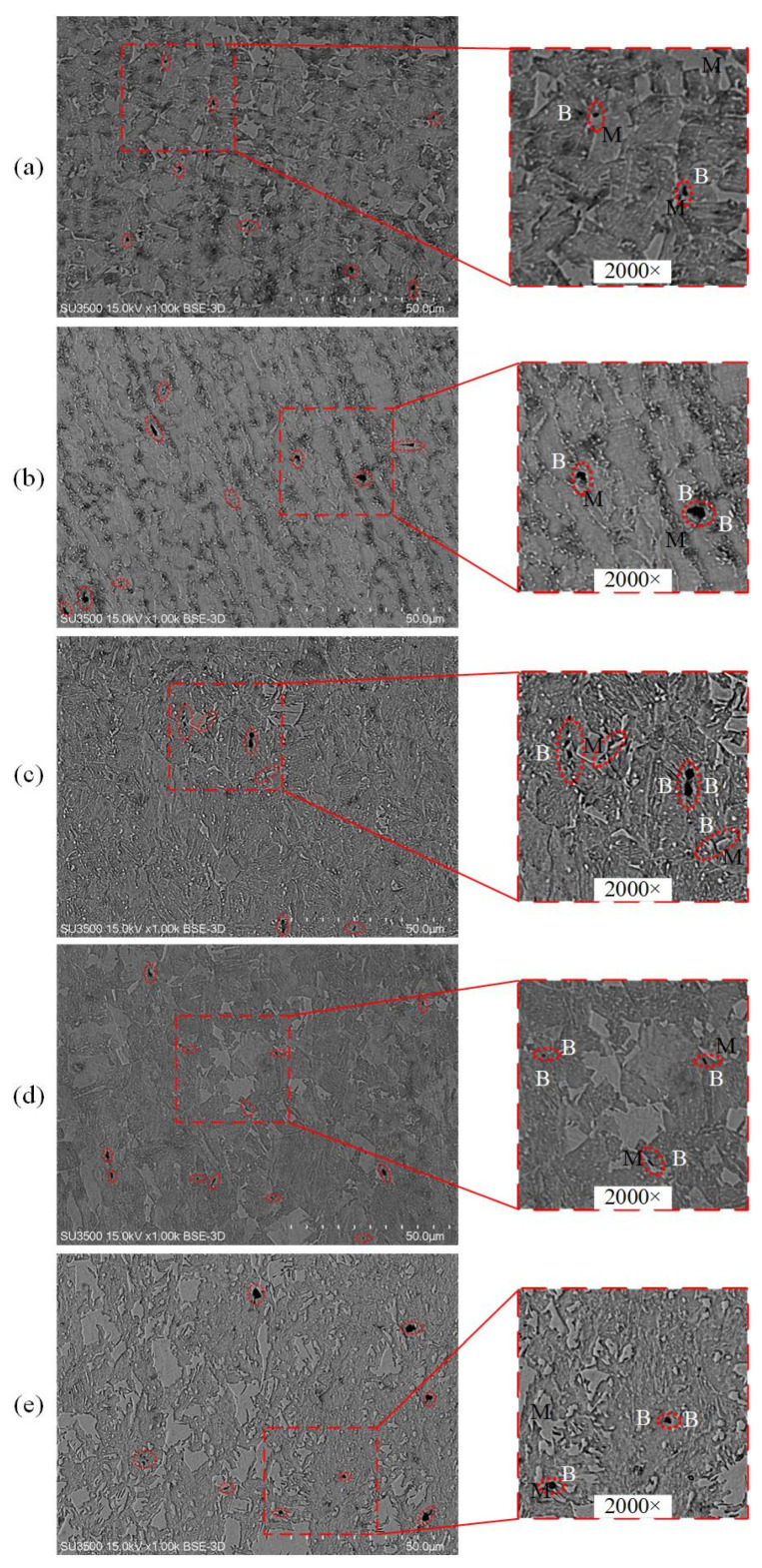
SEM micrographs showing void initiation and crack growth for 80%B + 20%M specimens under varying stress states: (**a**) pure shear specimen; (**b**) dogbone uniaxial tension specimen; (**c**) uniaxial tension specimen with a central hole; (**d**) plane strain tension specimen; (**e**) equi-biaxial tension specimen.

In order to obtain the evolution of the voids in the microstructure before fracture, SEMs were performed near the fractured surface after tensile tests. Figure 21 and Figure 22 illustrate void initiation and micro-crack propagation under different stress states for 30%B + 70%M and 80%B + 20%M, respectively. From the Figures, it can be found that there are three types of void initiation: The first type (type I) occurs at the phase boundary between bainite and martensite due to the deformation mismatch between the two phases, as shown in Figure 21a,c–e and Figure 22a,b,d. The second type (type II) is notified in the bainite phase shown in Figure 22c,e. With the growth of deformation bands, the martensite phase cannot bear plastic deformation anymore [49], and the third type of void initiation (type III) forms due to the failure in the martensite phase, as illustrated in Figure 21b. In Figure 21, the microstructure is martensite as matrix and bainite as inclusion, and martensite forms a continuous network around bainite grains. Type I and type III void nucleation modes were found. Under uniaxial tensile loading, martensite cracking can be clearly seen, while under shear, plane strain, and equi-biaxial tensile loading, a large number of voids located in the B/M boundary were found. As shown in Figure 22, the microstructure morphology is bainite as matrix and martensite as inclusion, and martensite phases are segmented by bainite phases and small martensite islands are formed. Type I void nucleation mode plays an important role under different stress states. It causes micro-cracks to initiate through two mechanisms. The first micro-crack grows along the interface and eventually transforms to a long strip of crack, as displayed in Figure 21c,e. The second micro-crack grows into the bainite grain to become a void, as shown in Figure 22b. Type II and type III void initiation models are prone to propagate towards the interior of bainite and martensite phases.

**Figure 23:**
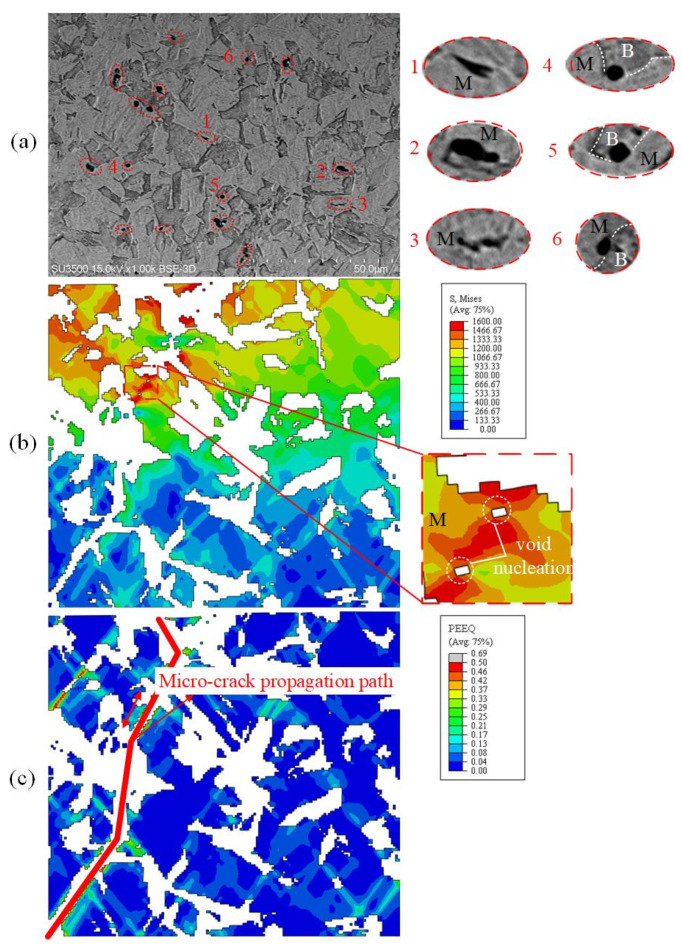
Comparison between SEM and simulation of 30%B + 70%M uniaxial tensile specimens: (**a**) SEM micrographs of damage mechanisms; (**b**) von Mises equivalent stress during void nucleation; (**c**) PEEQ at complete fracture.

**Figure 24:**
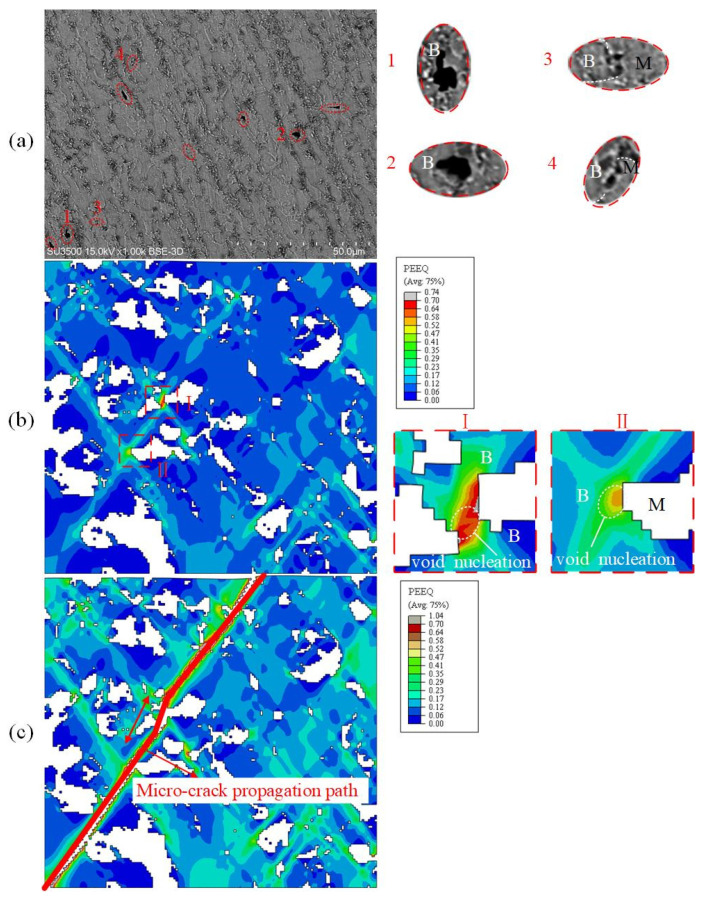
Comparison between SEM and simulation of 80%B + 20%M uniaxial tensile specimens: (**a**) SEM micrographs of damage mechanisms; (**b**) PEEQ during void nucleation; (**c**) PEEQ at complete fracture.

To further explore the void nucleation mode and micro-crack propagation mechanism, uniaxial tensile specimens with different B/M volume phase fractions were analyzed comparatively. The 30%B + 70%M and 80%B + 20%M specimens with the holding time of 10 s and 30 s, respectively, were selected. The similarity between the two microstructures is that a void initiates at the B/M boundary (Figure 23a, regions 4–6 and Figure 24a, regions 3–4). The B/M boundary possesses a large strain gradient, as shown in Figure 24b region Ⅱ, which is induced by the uneven deformation between the two phases. The difference is that the microstructure of 30%B + 70%M is mainly martensite and void nucleation is inclined to occur inside the martensite phase, while for the 80%B + 20%M microstructure dominated by bainite, void nucleation tends to occur in the bainite phase. This phenomenon has been verified in Figure 23a, regions 1–3 and Figure 24a, regions 1–2. The nucleation mechanism of these two kinds of voids is different. Figure 23b exhibits the distribution of von Mises equivalent stress of martensite in RVE, indicating that the stress distribution is not uniform during the deformation process, and the voids are prone to initiate under high stress states. This result is consistent with Ref. [25]. Figure 24b shows the PEEQ distribution in bainite at the beginning of damage. The void nucleation is in the bainite phase surrounded by martensite, and the strain value is significantly higher than that in other zones, as shown in Figure 24b region I;. The results indicate that the void nucleation in bainite is caused by high strain. In addition, the propagation mode of micro-cracks is also different with different volume fractions of martensite. Micro-cracks will propagate through martensite phase at high volume fractions of martensite, as shown in Figure 24c. As the volume fraction of martensite decreases to about 20%, the micro-cracks propagate near the B/M boundary, rather than through the martensite phase.

**Figure 25:**
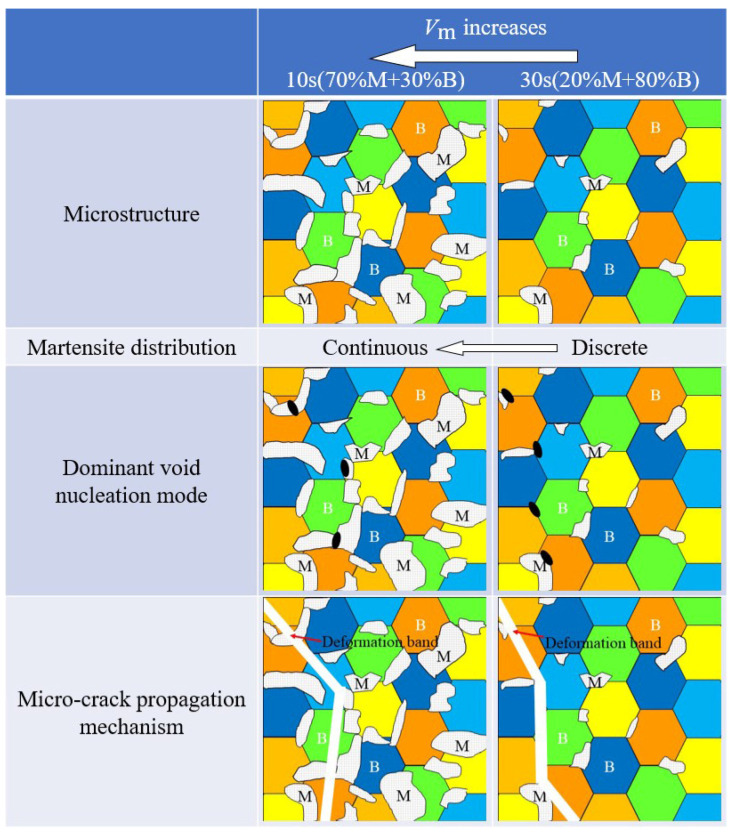
Schematic diagram of the connections among the Vm, martensite distribution, dominant void nucleation mode, and micro-crack propagation mechanism.

As shown in Figure 25, in case of a large volume fraction of martensite (30%B + 70%M), where the bainite phases are totally surrounded by network of martensite microstructure and the shear bands are forced to propagate through the martensite. In case of low volume fraction of martensite, since martensite islands are discretely distributed, their ability to resist the deformation of bainite is weak. In this case, the shear bands of bainite can be connected almost straightly without distortion. In short, as martensite distribution continuously changes from discrete to continuous, the dominant mode of damage nucleation in the mixed microstructure changes from B/M decohesion to martensite cracking.

## 5. Conclusions

Pure martensite, pure bainite, and B/M microstructures were generated by two-step quenching process. Constitutive equations of bainite and martensite phase were obtained by the reverse engineering approach. The effective stress–strain curves of specimens with different volume fractions of B/M structures are depicted by using two-dimensional RVE micromechanics method, which is consistent with tensile tests. The fracture strains of bainite and martensite in different stress states were determined by the hybrid numerical–experimental approach. The 3D damage curves of the individual phases were established based on the Lou-Huh ductile fracture criterion in the coordinate of the stress triaxiality, the Lode parameter, and fracture strain, and was used to calculate the damage curve of 80%B + 20%M boron steel specimens using RVE. The predicted damage curve is consistent with the fracture strain under varying stress states.

Three types of void nucleation modes under different stress states were explored: void initiation at the boundary of B/M, in bainite phase, and in martensite phase. Voids tend to initiate at the B/M boundary in the case of low stress triaxialities and in bainite at intermediate and high stress triaxialities for 80%B + 20%M microstructures. With the increase of Vm, the martensite distribution changes from discrete to continuous, followed by the nucleation location of voids moving from bainite to martensite. The ductile damage is caused by the incompatible deformation behavior of the B/M phases, resulting in severe plastic strain localization at the interface of the two phases. When the microstructure is dominated by bainite phase, it can bear greater plastic deformation. Under the influence of high strain, voids are more likely to be generated in the bainite phase, and the deformation zone is confined to the bainite channel between martensite islands. When the microstructure is dominated by the martensite phase, voids in the martensite phase are attributed to high stress and deformation bands will pass through martensite phases.

## Figures and Tables

**Table 1:** The composition percentages of the as-delivered 22MnB5 boron steel sheet (in wt. %).

C	Si	Mn	P	S	Cr	Ti	B
0.21	0.25	1.34	0.009	0.006	0.20	0.03	0.002

**Table 2:** The comparison of phase volume fractions of 22MnB5 samples with varying holding time by two-stage color tint-etching approach and SEM.

Time (s)	Modified LePera		SEM	
	Martensite (%)	Bainite (%)	Martensite (%)	Bainite (%)
10	70	30	64	36
20	32	68	34	66
30	20	80	18	82
40	10	90	12	88
50	4	96	5	95

**Table 3:** The crosshead velocity for different specimens in order to obtain quasi-static loading conditions (mm/min) [15].

Dogbone	Central Hole	Notched	In-Plane Shear	Nakajima
1.8	0.3	0.5	0.5	30

**Table 4:** Material parameters to predict the phase flow curves of pure bainite and martensite specimens, respectively.

Phase	$\sigma_{0}\;(\mathrm{MPa})$	Δσ (MPa)	α	M	b (m)	μ	*k*	$L_{k}\;(m)$
Bainite	218.29	360.97	0.33	3	${2.5\;\times \;10}^{-10}$	80	5.75	${3.24\;\times \;10}^{-6}$
Martensite	218.29	834.73	0.33	3	${2.5\;\times \;10}^{-10}$	80	16.75	${3.6\;\times \;10}^{-7}$

**Table 5:** Determined constants used for prediction the flow curves of the specimens with varying bainite volume fractions.

Phase Fraction	Δσ (MPa)	*k*	$L_{k}\;(m)$
B30	555.4	0.644	${2.213\;\times \;10}^{-6}$
B68	484.8	1.958	${2.288\;\times \;10}^{-6}$
B80	400.4	2.7	${3.38\;\times \;10}^{-6}$
B90	390.4	4.4	${2.9\;\times \;10}^{-6}$
B96	370.4	5.6	${3\;\times \;10}^{-6}$

**Table 6:** Determined constants used for prediction the flow curves of the specimens with varying martensite volume fractions.

Phase Fraction	Δσ (MPa)	*k*	$L_{k}\;(m)$
M4	1034.73	16.75	${3.6\;\times \;10}^{-7}$
M10	994.73	16.75	${3.6\;\times \;10}^{-7}$
M20	964.73	16.75	${3.6\;\times \;10}^{-7}$
M32	934.73	16.75	${3.6\;\times \;10}^{-7}$
M70	864.73	16.75	${3.6\;\times \;10}^{-7}$

**Table 7:** Fracture stain, the Lode parameter, and the stress triaxiality obtained by hybrid numerical–experimental approach for bainite specimens.

Specimen	Lode Parameter	Lode Angle	Normalized Lode Angle	Stress Triaxiality	Fracture Strain
Central hole	−0.4775	0.2546	0.5138	0.7169	0.9901
Notched R5	−0.0458	0.4972	0.0505	0.7527	0.8896
In-plane shear	−0.0945	0.4691	0.1041	0.0365	1.1766
Nakajima	0.9469	1.0239	−0.9555	0.6603	0.8719

**Table 8:** Fracture stain, the Lode parameter, and the stress triaxiality obtained by hybrid numerical–experimental approach for martensite specimens.

Specimen	Lode Parameter	Lode Angle	Normalized Lode Angle	Stress Triaxiality	Fracture Strain
Central hole	−0.8030	0.0895	0.8291	0.5290	0.6458
Notched R5	−0.0537	0.4926	0.0592	0.6892	0.5155
In-plane shear	−0.0517	0.4937	0.0570	0.0211	0.9076
Nakajima	0.9780	1.0376	−0.9817	0.6578	0.3612

**Table 9:** The values of applied uniform loads $F_{1}\;$ and $F_{2}$ for different stress triaxialities.

$F_{1}\;(N).$	$F_{2}\;(N)$	$F_{2}$ $/F_{1}$	Stress Triaxiality
100	−80	−0.8	0.02
100	0	0	0.33
100	18	0.18	0.44
100	60	0.6	0.57
100	100	1	0.63

**Table 10:** Fracture stains, the Lode parameter, and the stress triaxiality obtained by hybrid numerical-experimental approach for 80%B + 20%M specimens.

Specimen	Lode Parameter	Lode Angle	Normalized Lode Angle	Stress Triaxiality	Fracture Strain
Central hole	−0.5738	0.2037	0.6110	0.6635	0.8608
Notched R5	−0.0465	0.4968	0.0513	0.7549	0.7835
In-plane shear	−0.0766	0.4794	0.0844	0.0294	1.0672
Nakajima	0.9243	1.0138	−0.9362	0.6564	0.5019

## Data Availability

The raw/processed data required to reproduce these findings cannot be shared at this time as the data also forms part of an ongoing study.
